# The Endocranial Anatomy of Therizinosauria and Its Implications for Sensory and Cognitive Function

**DOI:** 10.1371/journal.pone.0052289

**Published:** 2012-12-19

**Authors:** Stephan Lautenschlager, Emily J. Rayfield, Perle Altangerel, Lindsay E. Zanno, Lawrence M. Witmer

**Affiliations:** 1 School of Earth Sciences, University of Bristol, Bristol, United Kingdom; 2 National University of Mongolia, Ulaanbaatar, Mongolia; 3 Nature Research Center, NC Museum of Natural Sciences, Raleigh, North Carolina, United States of America; 4 Department of Biology, North Carolina State University, Raleigh, North Carolina, United States of America; 5 Department of Biomedical Sciences, Heritage College of Osteopathic Medicine, Ohio University, Athens, Ohio, United States of America; Ludwig-Maximilians-Universität München, Germany

## Abstract

**Background:**

Therizinosauria is one of the most enigmatic and peculiar clades among theropod dinosaurs, exhibiting an unusual suite of characters, such as lanceolate teeth, a rostral rhamphotheca, long manual claws, and a wide, opisthopubic pelvis. This specialized anatomy has been associated with a shift in dietary preferences and an adaptation to herbivory. Despite a large number of discoveries in recent years, the fossil record for Therizinosauria is still relatively poor, and cranial remains are particularly rare.

**Methodology/Principal Findings:**

Based on computed tomographic (CT) scanning of the nearly complete and articulated skull of *Erlikosaurus andrewsi*, as well as partial braincases of two other therizinosaurian taxa, the endocranial anatomy is reconstructed and described. The wider phylogenetic range of the described specimens permits the evaluation of sensory and cognitive capabilities of Therizinosauria in an evolutionary context. The endocranial anatomy reveals a mosaic of plesiomorphic and derived characters in therizinosaurians. The anatomy of the olfactory apparatus and the endosseous labyrinth suggests that olfaction, hearing, and equilibrium were well-developed in therizinosaurians and might have affected or benefited from an enlarged telencephalon.

**Conclusion/Significance:**

This study presents the first appraisal of the evolution of endocranial anatomy and sensory adaptations in Therizinosauria. Despite their phylogenetically basal position among maniraptoran dinosaurs, therizinosaurians had developed the neural pathways for a well developed sensory repertoire. In particular olfaction and hearing may have played an important role in foraging, predator evasion, and/or social complexity.

## Introduction

Therizinosauria comprises an enigmatic clade of Cretaceous (Barremian – Maastrichtian) maniraptoran dinosaurs found in Asia and North American [1]. They are distinguished from other theropods by an unusual suite of morphological characters, which include lanceolate, tightly packed teeth, a rostral rhamphotheca, an elongate neck, enlarged manual claws, and a wide, opisthopubic pelvis. Many of these characters have been associated with an herbivorous diet and specializations in foraging behavior [2]. Recent phylogenetic analyses [3], [4] have recovered Therizinosauria close to the base of Maniraptora. Nested between basal coelurosaurs and derived maniraptorans, this makes them of special importance not only in terms of the evolution of anatomical and developmental patterns, but also for the radiation of herbivory among theropods [5]. Their unique morphology has resulted in a plethora of hypotheses on the evolution, dietary preferences, and behavior of therizinosaurians – most of which have been inferred, based on skeletal traits and anatomical characters [6]–[9]. However, the endocranial anatomy has rarely been considered, and little is known about the sensory and cognitive abilities in therizinosaurians and whether any of these reflect adaptations to an herbivorous lifestyle.

As the neurocranium and the brain are closely associated during growth, the endocranial cavity constitutes a representation of the gross surficial structure of the brain itself. A cast of the endocranial cavity, thus can provide the means to reconstruct the brain anatomy and associated structures [10], [11]. The neural centers for the senses of vision, olfaction, hearing and balance either reside in the brain or peripherally in the inner ear, allowing us to evaluate the relative sensory capabilities using a series of qualitative and quantitative measures.

A variety of cranial endocasts (both digital and physical) of dinosaurs in general have been described in recent years [12]–[15]. For theropods, these include mainly basal and non-maniraptoriform taxa [16]–[23], probably in part because braincase elements are more likely to be preserved in larger animals. In contrast, data on brain anatomy and endocasts are rare in Maniraptoriformes and restricted to some scattered specimens among the individual lineages [24]–[27]. Given the paucity of available material, it may not be surprising that only a handful of studies focus on a broader evolutionary context of brain anatomy and sensory adaptations [10], [14], [21], [28], [29], [30].

Although cranial remains are still rare, numerous discoveries have expanded our knowledge of therizinosaurians in the last 20 years. To date, the holotype of *Erlikosaurus andrewsi* (IGM 100/111) from the Late Cretaceous of Mongolia includes the only complete, articulated, and three-dimensionally preserved skull (including the braincase) of any therizinosaurian [31]. However, partial braincases are known for the therizinosaurid *Nothronychus mckinleyi* (AZMNH-2117) from the Upper Cretaceous (Turonian) Moreno Hill Formation of New Mexico [32], [33] and the basal therizinosaurian *Falcarius utahensis* (UMNH VP 15000 & 15001) from the Lower Cretaceous (Barremian) Cedar Mountain Formation of Utah [34], [35].

**Figure 1:**
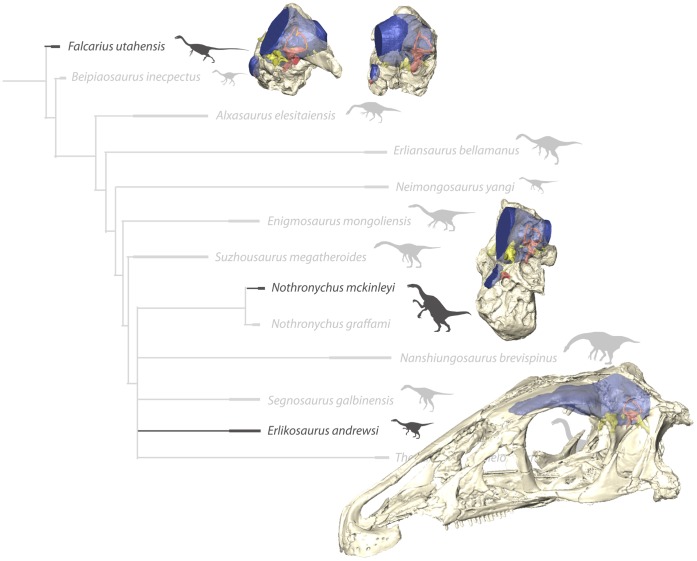
In-situ endocranial elements of the described therizinosaurian taxa displayed in their phylogenetic context. Bone rendered transparent to reveal endocranial anatomy. (Phylogeny modified from [4]).

Based on the endocasts of these four specimens, we present an extensive description of the endocranial anatomy of therizinosaurians (Figure 1). The wide phylogenetic range of these samples, spanning both basal and derived taxa and thus a considerable part of the known therizinosaurian diversity, allows inferences to be drawn about sensory and cognitive capabilities. It further provides the foundation for the study of endocranial and sensorineural evolution in Therizinosauria. This is potentially relevant to the discussion of whether therizinosaurian sensory (and cognitive) abilities were specially adapted to herbivory.

## Materials and Methods

### Specimens and Computed Tomography (CT)

The skull of *Erlikosaurus andrewsi* (IGM 100/111) was CT scanned at X-Tek Systems Ltd. (now Nikon Metrology), Tring, Hertfordshire, using a XT-H-225ST CT scanner fitted with a special steel honeycomb support to minimize object movement. Scan parameters were set at 180 kV and 145 µA for the complete skull. Additional scans were performed for the braincase region at 180 kV and 135 µA. The resulting rotational projections were processed with custom-built software provided by X-Tek Systems Ltd. creating.vgi and.vol files containing 1998 slices with a slice thickness of 145 µm for the complete skull and 1000 slices with a slice thickness of 108 µm for the braincase region.

CT scans of two partial braincases of *Falcarius utahensis* (holotype specimen UMNH VP 15000, referred specimen UMNH VP 15001) and a partial braincase of *Nothronychus mckinleyi* (AZMNH-2117) were performed at Ohio University using a General Electric eXplore Locus in vivo Small Animal MicroCT Scanner at 80 kV and 498 µA. Scans of the holotype and referred specimens of *Falcarius* yielded 781 and 960 slices, respectively, both with a slice thickness of 92 µm, whereas the *Nothronychus* scans yielded 1008 slices with a slice thickness of 88 µm. The respective scan data are part of an ongoing doctoral dissertation of the first author and will be deposited in an electronic archive and with the respective museum collections after completion of the dissertation.

The final data files were subsequently imported into Avizo (Version 6.3.1., Visualization Science Group), running on a Hewlett-Packard workstation with 72 GB RAM and an nVidia Quadro 4000 2 GB graphics card under Windows 7 Professional (64 bit). Anatomical structures of interest (endocasts, endosseous labyrinths, cranial nerves, and blood vessels) were highlighted and separately labeled using Avizo's segmentation editor. 3D surface models and volumes were created to visualize the highlighted structures and illustrate this article. Additionally, all measurements presented in this article and used for various calculations were derived from the digital models using Avizo (see Table 1). A 3d pdf document with interactive figures can be found in supplementary information.

**Table 1:** Linear and volumetric dimensions of the discussed endocasts and endosseous labyrinths**.**

	*Erlikosaurus andrewsi*	*Nothronychus mckinleyi*	*Falcarius utahensis*(UMNH VP 15000)	*Falcarius utahensis* (UMNH VP 15001)
Endocast length (includingolfactory apparatus) [mm]	101	–	–	–
Olfactory apparatus length [mm]	36	–	–	–
Maximum width of cerebralhemispheres [mm]	38	–	–	–
Maximum height of cerebralhemispheres [mm]	20	–	–	–
Maximum length of cerebralhemispheres [mm]	30	–	–	–
Endocast volume (as preserved)[mm3]	34 118	11 526	19 561	23 437
Cerebral hemispheres volume[mm^3^]	13 958	–	–	–
Cochlear duct length [mm]	11.06	9.93 (right)	12.85 (left)/13.79 (right)	13.80 (left)/11.92 (right)

Due to some extensive damage to the caudal left braincase wall (basisphenoid, laterosphenoid, prootic, exoccipital) of *Erlikosaurus*, it was not possible to completely reconstruct this part of the endocast. To obtain a complete endocast, the individual slices of the data set were mirrored and the missing part of the endocast was created and registered for the left side separately. To automate these steps two tcl (tool command language) scripts were created for Avizo. The endocast description is therefore mostly based on the original right side, unless specified otherwise. This method was also applied to acquire and supplement data for some parts of the endosseous labyrinths of *Falcarius* and *Nothronychus*. Following Sampson and Witmer [20], we will use the term cranial endocast in reference to the space within the braincase, housing the actual brain, the meninges and dural venous sinuses, the cerebral blood vessels, and the cranial nerve roots. To facilitate the discussion of these elements, we will further refer to the digital cast of the structures as if they were the structures themselves. We focus our description on *Erlikosaurus andrewsi*, as its endocast is the most complete and shows the majority of anatomical features. The phylogenetic and taxonomic classifications follow Zanno [4], by using the definitions for Therizinosauria and correspondingly therizinosaurian, to describe a clade including the basal taxon *Falcarius utahensis*, whereas the terms Therizinosauroidea and therizinosauroid, and Therizinosauridae and therizinosaurid define more exclusive clades within Therizinosauria.

No permits were required for the described study, which complied with all relevant regulations.

### Body-mass Estimations

Relative and correlated measures of sensory capabilities are typically scaled to body mass. In the case of therizinosaurians, the general lack of preserved fossil remains usable for body-mass calculations poses a considerable problem. Moreover, the exceptional body morphology and proportions, which differ from those of other theropods, make comparisons to better-known theropod groups difficult. For example, although Therrien and Henderson [36] developed a method to calculate body length and body mass from skull length in theropods, they only considered “strictly carnivorous, exclusively terrestrial, non-avialian theropod taxa,” excluding ornithomimosaurs, oviraptorids, and therizinosaurians due to their disproportionally small skulls.

Of the three therizinosaurian taxa discussed here, the only published mass estimate was given by Russell and Dong [37] for *Erlikosaurus andrewsi*, who estimated its body mass at approximately 160 kg. This value was obtained by calculating a mass estimate of 380 kg for the more complete *Alxasaurus elesitaiensis* using Anderson et al.’s [38] relationship of femur circumference and body weight, and then by downscaling it for *Erlikosaurus*, by applying the principle of geometrically similar bodies [39].

Here we use theropod-specific equations relating body mass to femoral length (FL) [40] to produce mass estimates for *Erlikosaurus*, *Nothronychus mckinleyi*, and *Falcarius* (Table 2). The largest femur from the holotype locality of *Falcarius utahensis* (UMNH 13423) measures 403 mm, yielding an estimated mass of 128 kg. However, femora are unknown for *Erlikosaurus* and *Nothronychus mckinleyi*. In order to estimate femoral length for these taxa, we performed bivariate regression analyses on log-transformed data to describe the relationship between femoral length (FL) and humeral length (HL) in six therizinosauriod taxa (*Nothronychus graffami*, *Alxasaurus elesitaiensis*, *Erliansaurus bellamanus*, *Neimongosaurus yangi*, and two undescribed taxa, Y. Kobayashi unpublished data). Although not a weight-bearing element, we chose the humerus because, among the fragmentary materials known for *Erlikosaurus* [41], it has the largest representation across Therizinosauria. Despite being small in number, the sampled taxa bracket the humeral lengths of *Erlikosaurus* and *Nothronychus mckinleyi* allowing interpolated prediction of FL and minimizing potential error. The resulting equation (log_10_ HL = −0.2218±0.2753+1.158±0.1116× log_10_ FL) produced a high correlation coefficient (r = 0.96). Applying the maximum humeral length of 300 mm for *Erlikosaurus* given by Perle [41], yields an estimated FL of 443.3 mm and a mass estimate of 174 kg, comparable to that obtained by Russell and Dong [37]. A humeral length of 418 mm for *Nothronychus mckinleyi*, results in a FL of 651 mm and a mass value of 599 kg. Given the large uncertainty inherent in our approach and in body-mass estimates in general, we assume a range of 150–250 kg for *Erlikosaurus* and 550–650 for *Nothronychus mckinleyi* (see also Table 2).

**Table 2:** Calculated values for femur length (FL) and body mass.

	Humerus length (HL) [mm]	Femur length (FL) [mm]	Body mass [kg]
*Erlikosaurus andrewsi*	300	443.3	173.7
*Falcarius utahensis*		403	127.7
*Nothronychus mckinleyi*	418	650.9	598.6

Based on bivariate regression analyses on log-transformed data for femoral length (FL) and humeral length (HL) (log_10_ HL = −0.2218±0.2753+1.158±0.1116 × log_10_ FL).

### Institutional Abbreviations

AZMNH, Arizona Museum of Natural History, Mesa, Arizona, USA.

IGM, Geological Institute of the Mongolian Academy of Sciences, Ulaan Bataar, Mongolia.

UMNH, Natural History Museum of Utah, Salt Lake City, USA.

## Results

### Cranial Endocast

**Figure 2:**
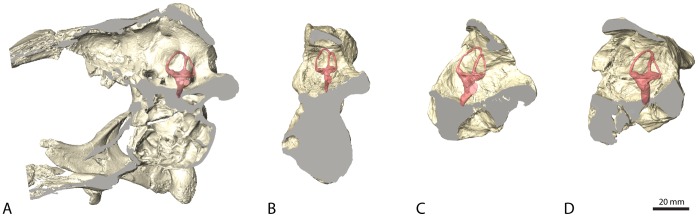
Sagittal sections through the individual braincases. (A*) Erlikosaurus andrewsi* (IGM 100/111), (B) *Nothronychus mckinleyi* (AZMNH-2117), (C) *Falcarius utahensis* (holotype specimen, UMNH VP 15000), (D) *Falcarius utahensis* (referred specimen, UMNH VP 15001), all in medial view. All braincases are oriented with the lateral semicircular canal aligned horizontally, as indicated by the position of the left endosseous labyrinth. The sagittal sections show the variable degree of endocast angulation and flexure caused by the outline of the braincase floor and the position of the foramen magnum.

The cranial endocasts of all four examined specimens are very similar in their general morphology, at least as far as can be determined from those parts that are preserved. The full extent of the endocast could only be reconstructed for *Erlikosaurus andrewsi*. Its endocast is relatively elongate and straight. The individual parts of the brain (fore-, mid- and hindbrain) are arranged in the same horizontal plane and do not show an obvious angulation, indicating only slight cephalic and pontine flexures. This is in contrast to more basal theropods, where the angulation of the midbrain can be up to 45–60 degrees [20]–[22], but also in contrast to the endocast of the holotype of *Falcarius utahensis* (UMNH VP 15000). Although incomplete, its morphology suggests a more pronounced angulation between the hindbrain and the midbrain. Smith et al. [35] remarked on the deep floor of the endocranial cavity, indicating the strong pontine flexure in *Falcarius*. However, this only applies to the holotype specimen (UMNH VP 15000) (Figure 2C). The excavation of the braincase floor and the corresponding angulation in the endocast are less pronounced in the referred specimen (UMNH VP 15001) of *Falcarius*. *Nothronychus* shows a more moderate flexure similar to *Erlikosaurus* (Figure 2B). However, as these reconstructions are based on partial braincases only, such morphological differences should be considered with caution.

**Figure 3:**
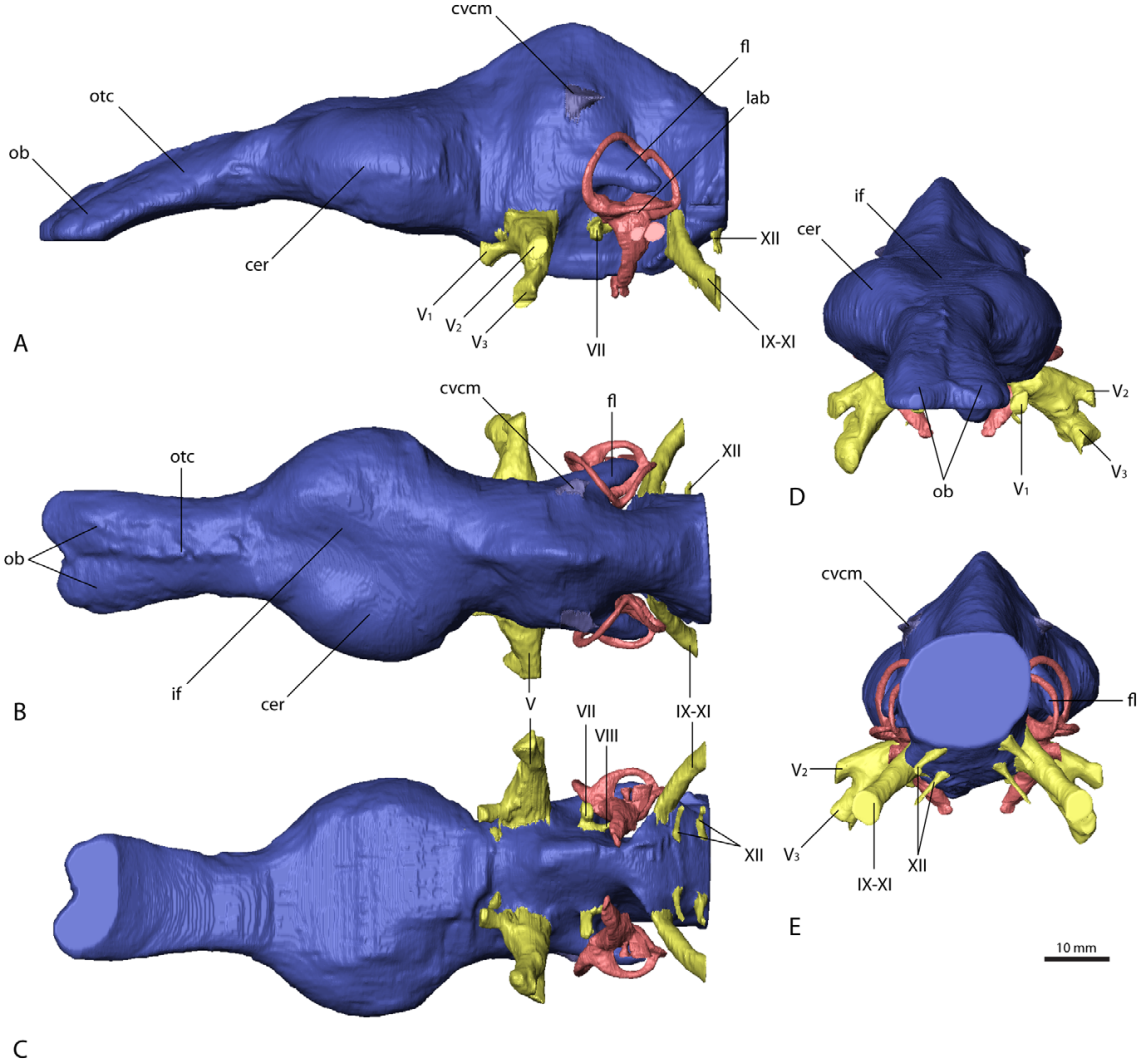
Cranial endocast of *Erlikosaurus andrewsi* (IGM 100/111). In (A) left lateral, (B) dorsal, (C) ventral, (D) rostral, and (E) caudal view. Abbreviations: cer, cerebral hemisphere; cvcm, caudal middle cerebral vein; fl, floccular lobe; if, interhemispherical fissure; lab, endosseous labyrinth; ob, olfactory bulbs; otc, olfactory tracts; V_1_, ophthalmic branch of the trigeminal nerve canal; V_2_, maxillary branch of the trigeminal nerve canal; V_3_, mandibular branch of the trigeminal nerve canal; VII, facial nerve canal; VIII, vestibulocochlear nerve canal; IX–XI, shared canal for the glossopharyngeal, vagus and spinal accessory nerve; XII, hypoglossal nerve canal.

**Figure 4:**
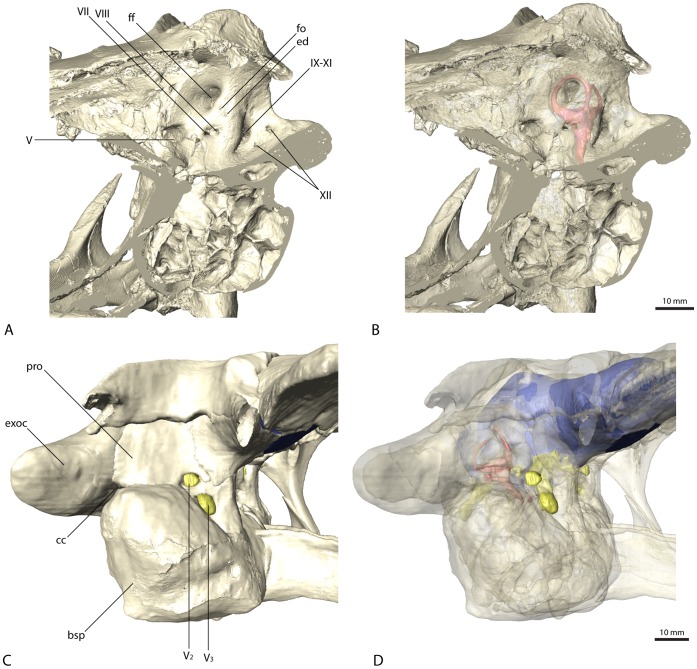
Braincase of *Erlikosaurus andrewsi* (IGM 100/111). Sagittal sections in (A) and (B) in rostromedial view. Lateral braincase wall in (C) and (D) in right lateral view with squamosal, postorbital, quadrate and quadratojugal digitally removed (based on a digital reconstruction of the cranial anatomy). Bone in (C) and (D) rendered transparent to reveal endocranial elements. Abbreviations: bsp, basisphenoid; cc, columellar canal; ed, endolymphatic duct; exoc, exoccipital and paroccipital process; ff, floccular fossa; fo, foramen; pro, prootic; V_2_, maxillary branch of the trigeminal nerve canal; V_3_, mandibular branch of the trigeminal nerve canal; VII, facial nerve canal; VIII, vestibulocochlear nerve canal; IX–XI, shared canal for the glossopharyngeal, vagus and spinal accessory nerve; XII, hypoglossal nerve canal.

In *Erlikosaurus* (Figure 3A–C), the forebrain region–most notably the olfactory apparatus and the cerebral hemispheres–is well differentiated and clearly demarcated. The olfactory tracts and bulbs are preserved only in *Erlikosaurus*. However, due to the lack of preserved or ossified sphenethmoid and mesethmoid elements [42] (compare Figure 4), the ventral extent of the olfactory apparatus is uncertain and has been reconstructed only tentatively as far as possible, tracing the ventral margins of frontal bones. The olfactory tracts are elongate and extend relatively far from the cerebral hemispheres and the actual brain. They are slightly downturned, following the shape of the skull roof. The olfactory bulbs are only moderately indicated by shallow impressions on the ventral surface of the frontals. They are of moderate size, as in other Maniraptoriformes (*Struthiomimus altus*, *Deinonychus antirrhopus*), for which endocranial casts are available [21]. In *Falcarius*, two incomplete frontals (UMNH VP 14524, 12525; [43]) show preserved, although only partial, impressions of olfactory tracts. Olfactory bulb size and tract length cannot be ascertained, but a considerable expansion of the olfactory apparatus is evident.

The cerebral hemispheres in *Erlikosaurus* are relatively large and are broader transversely than the caudal part of the endocast. Their contours are clearly reproduced by the endocranial cavity for the entire hemispheres, indicating a close association between the brain and the endocranial wall and the presence of only a thin dural envelope between the two. Complex vascular grooves on the endocranial surface, typically found in birds and mammals [44], but also in some ornithischians [45], tyrannosaurs ([21], respective figure 7), oviraptorids [46] and troodontids [47], are not observed in *Erlikosaurus*. A pair of semi-articulated frontals referred to *Falcarius utahensis* (UMNH VP 14524, 12525; [43]) shows pronounced endocranial impressions. Although only incompletely preserved, their size indicates equally enlarged cerebral hemispheres as in *Erlikosaurus*. In the latter, the cerebral hemispheres are separated dorsally by an interhemispherical fissure (Figure 3C).

Optic lobes or tecta are not visible in the endocast of *Erlikosaurus*. The enlarged cerebral hemispheres would suggest that the optic lobes are situated in a ventrolateral position caudal to the cerebral hemispheres and rostral to the transverse sinus [21]. However, surface structures in this region are not clear enough to distinctly demarcate the optic lobes. Pronounced and ventrolaterally displaced optic lobes are considered to reflect a more derived organization of the brain. Within coelurosaurs, and especially Maniraptoriformes, there is a clear trend for the expression of this feature [10], [21], [29]. Given that more basal coelurosaurs such as ornithomimosaurs also display the derived condition, it is likely that therizinosaurians also had ventrolaterally displaced optic lobes that were concealed within the dura, but we cannot rule out that they secondarily acquired the primitive, more dorsal position or were simply too small to make their position apparent.

**Figure 5:**
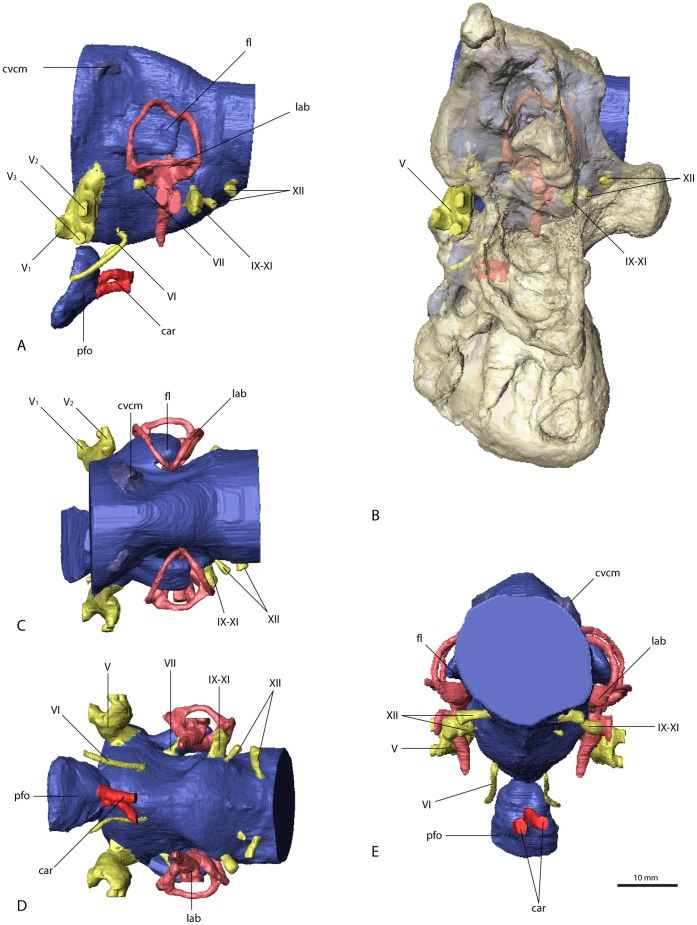
Cranial endocast and braincase of *Nothronychus mckinleyi* (AZMNH-2117). In (A) and (B) in left lateral, (C) caudal, (D) dorsal, and (E) ventral view. Bone in (B) rendered transparent. Abbreviations: car, cerebral carotid artery canal; cvcm, caudal middle cerebral vein; fl, floccular lobe; lab, endosseous labyrinth; pfo, pituitary (hypophyseal) fossa; V_1_, ophthalmic branch of the trigeminal nerve canal; V_2_, maxillary branch of the trigeminal nerve canal; V_3_, mandibular branch of the trigeminal nerve canal; VI, abducens nerve canal; VII, facial nerve canal; IX–XI, shared canal for the glossopharyngeal, vagus and spinal accessory nerve; XII, hypoglossal nerve canal.

**Figure 6:**
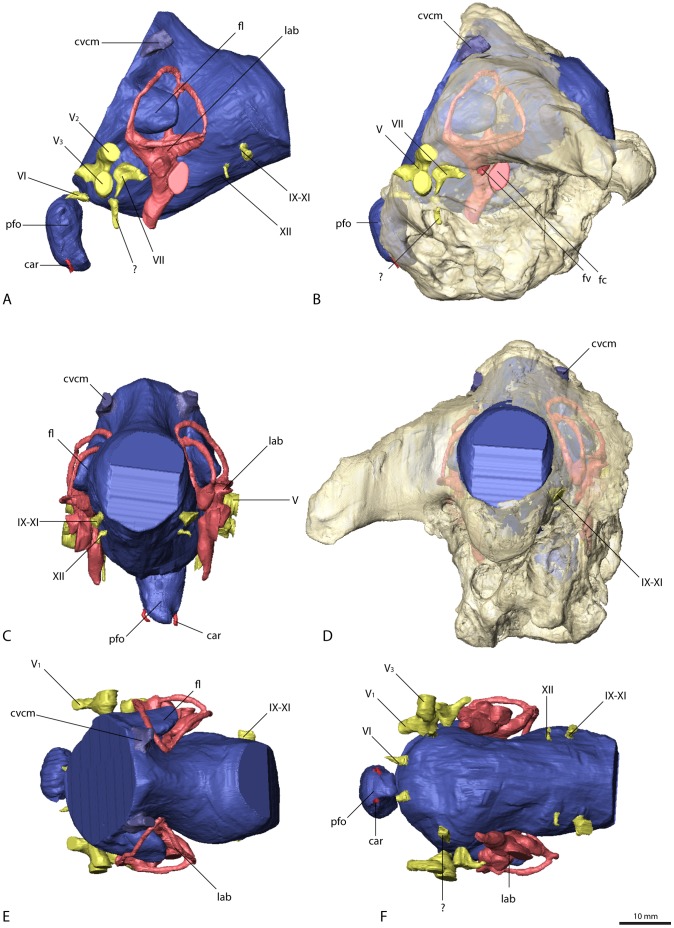
Cranial endocast and braincase of *Falcarius utahensis* (holotype, UMNH VP 15000). In (A) and (B) in left lateral, (C) and (D) in caudal, (E) dorsal, and (F) ventral view. Bone in (B) and (D) rendered transparent. Abbreviations: car, cerebral carotid artery canal; cvcm, caudal middle cerebral vein; fc, fenestra cochleae; fl, floccular lobe; fv, fenestra vestibuli; lab, endosseous labyrinth; pfo, pituitary (hypophyseal) fossa; V_1_, ophthalmic branch of the trigeminal nerve canal; V_2_, maxillary branch of the trigeminal nerve canal; V_3_, mandibular branch of the trigeminal nerve canal; VI, abducens nerve canal; VII, facial nerve canal; IX–XI, shared canal for the glossopharyngeal, vagus and spinal accessory nerve; XII, hypoglossal nerve canal.

**Figure 7:**
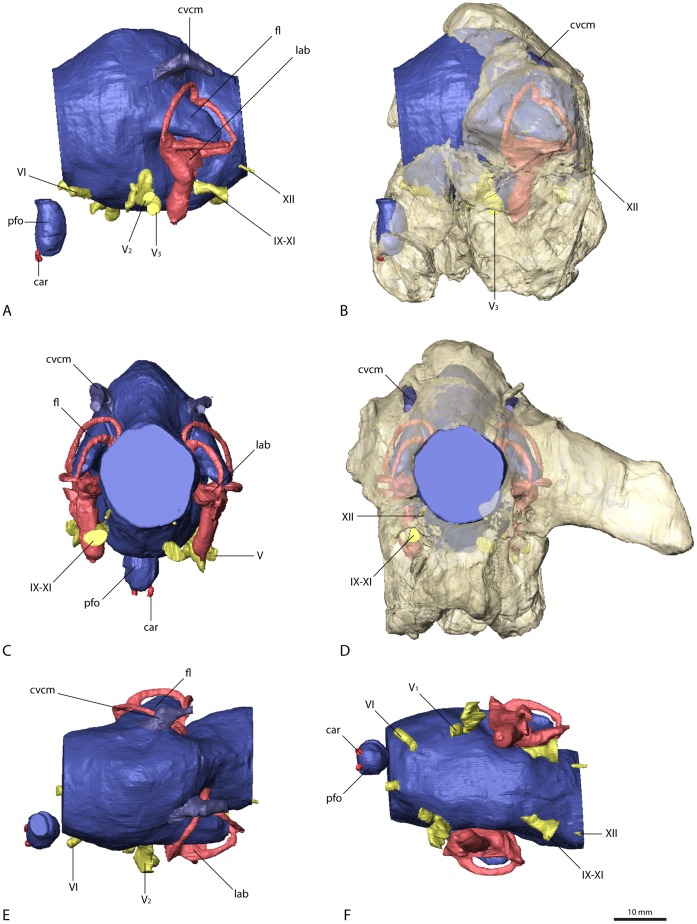
Cranial endocast and braincase of *Falcarius utahensis* (referred specimen, UMNH VP 15001). In (A) and (B) in left lateral, (C) and (D) in caudal, (E) dorsal, and (F) ventral view. Bone in (B) and (D) rendered transparent. Abbreviations: car, cerebral carotid artery canal; cvcm, caudal middle cerebral vein; fl, floccular lobe; lab, endosseous labyrinth; pfo, pituitary (hypophyseal) fossa; V_1_, ophthalmic branch of the trigeminal nerve canal; V_2_, maxillary branch of the trigeminal nerve canal; V_3_, mandibular branch of the trigeminal nerve canal; VI, abducens nerve canal; IX–XI, shared canal for the glossopharyngeal, vagus and spinal accessory nerve; XII, hypoglossal nerve canal.

The cerebellum is not as clearly demarcated in the endocast as is the cerebrum, but it appears to be relatively high and narrow in *Erlikosaurus* (Figure 3A, B), as well as in the other three therizinosaurian specimens (Figure 5, 6, 7). It is situated in the space between the endosseous labyrinths. The height, however, cannot be exactly determined, as a dural venous sinus covered the dorsal part of the endocast, obscuring cerebellar details. A moderately developed dural peak is present in all the therizinosaurian endocasts. Ventrally, the outline of the pons is indicated by an expanded area, which merges with the medulla oblongata. The latter is considerably enlarged and as wide as the cerebellum at its widest expansion, where it exits the braincase through the foramen magnum. Whereas the medulla oblongata is slightly wider than high in *Erlikosaurus* and *Nothronychus* (Figure 5E), it is slightly higher than wide in the two endocasts of *Falcarius* (Figure 6C, 7C).

The floccular lobes (cerebellar auricles) in all the therizinosaurian endocasts project caudolaterally from the cerebellar region and enter the space between the semicircular canals of the endosseous labyrinth. The floccular lobes are enlarged and have a round to oval cross-section in *Erlikosaurus*, whereas they are transversely flattened in *Falcarius* and *Nothronychus*. In *Falcarius*, the flocculi barely extend caudally beyond the crus communis (Figure 6A). They extend further in *Erlikosaurus* and *Nothronychus*, but do not pass beyond the lateral and caudal semicircular canals, filling the space between the canals to a large extent.

Due to the lack of preservation of bony correlates, the pituitary fossa could not be faithfully reconstructed for *Erlikosaurus*, and only partially for *Falcarius* and *Nothronychus*. It is most complete in one of the specimens of *Falcarius* (UCMP 15001), where it is dorsoventrally elongated and oval with a slight constriction dorsally. The canals for the cerebral carotid artery are positioned ventrally on each side of the pituitary fossa and are small in *Falcarius*. In *Nothronychus* they are enlarged and situated close together, projecting caudally.

### Cranial Nerves and Veins

The majority of the cranial nerves could be traced and visualized in all four described endocasts. The canals for the optic (CN II), oculomotor (CN III) and trochlear (CN IV) nerves could not be reconstructed for any of the discussed specimens. In *Erlikosaurus*, where the braincase is the most complete, the rostromedial parts of the laterosphenoids and orbitosphenoids are only fragmentarily preserved. In the partial braincases of *Falcarius* and *Nothronychus*, this region is completely missing. The canal for the trigeminal nerve (CN V) is large and originates from the endocast ventrolaterally of the cerebellum and between the abducens and facial nerve canal in *Erlikosaurus*. The trigeminal nerve canal splits up into a rostrally directed smaller ophthalmic branch (CN V_1_), which passes through a separate ophthalmic foramen, and a larger branch exiting the braincase laterally. The latter branch subsequently divides into a more dorsally oriented maxillary branch (CN V_2_) and a ventral mandibular branch (CN V_3_), each passing through a separate foramen. Due to preservation, the osteological correlates of the trigeminal nerve canals are less clear in *Nothronychus* and *Falcarius*. However, in both taxa a split of the trigeminal nerve into three separate branches as in *Erlikosaurus* is evident. The CT scans confirm the trigeminal foramen identified by Kirkland et al. [33] on the left side of the braincase in *Nothronychus* (Figure 5B). The single foramina of the separate branches are not preserved and therefore are not visible in the specimen on this side. The respective region is more complete but still largely encased in matrix on the right side. In the CT scans the three separate nerve canals could be identified, indicating that the large foramen on the left side is the exit of the nerve root. A putative foramen for the maxillary and mandibular branches suggested by Smith et al. [35] (identified as the abducens foramen by Kirkland et al. [33]) is a blind pocket or pneumatic space, with no connection to the endocranial cavity.

The abducens nerve (CN VI) canal is only incompletely preserved in *Erlikosaurus*. It originates ventrolaterally from the endocast rostromedial to the trigeminal nerve canal, passing through a medium sized abducens foramen in the orbitosphenoid. This part of the braincase is only fragmentarily preserved and for some parts crushed. Therefore the exact distal course of the abducens nerve canal cannot be traced with certainty, but the lateral position of both canals indicate that they did not pass through the pituitary fossa, which is a derived condition found in most coelurosaurs [21]. As in *Erlikosaurus*, the abducens nerve canal in *Falcarius* originates rostrally to the trigeminal nerve canal bypassing the pituitary fossa. Smith et al. [35] suggested that this canal probably also housed the oculomotor nerve (CN III), which would be very unusual; given that the oculomotor foramen tends to be more dorsally situated between the laterosphenoid and orbitosphenoid [11], [21], we regard the oculomotor foramen as not being preserved in the *Falcarius* or *Nothronychus* braincases. In *Nothronychus*, the abducens nerve canal originates from the endocast caudal to the trigeminal nerve canal. It exits the braincase rostrolaterally to the pituitary fossa without passing through the latter. The abducens foramen however is filled with matrix and not clearly visible in the actual specimen. The abducens foramen identified by Kirkland et al. [33] shows no connection to the endocranial cavity (see above).

A small canal caudal to the trigeminal nerve canal represents the facial nerve canal (CN VII) in *Erlikosaurus*. It originates from the endocast in close association with the vestibulocochlear nerve (CN VIII), but both canals are clearly separated. The facial nerve canal exits the braincase through a small foramen ventral to the floccular fossa and caudal to the large foramen for the trigeminal nerve canal (Figure 4A). Laterally, the facial nerve splits up into two small branches. The dorsal branch enters the vestibular eminence and contacts the endosseous labyrinth medially, rostral to the base of the crus communis, and therefore possibly represents the hyomandibular branch or parts of it. The more ventrally located branch of the facial nerve canal is larger than its dorsal counterpart and continues in ventrolateral direction. After exiting the braincase, its course cannot be traced further. Based on its position, it probably represents the palatine branch of the facial nerve.

In *Nothronychus* the facial nerve canal is comparable to that of *Erlikosaurus* in both location and size. A split into two branches is not visible. This is either due to the lower resolution of the CT scans and/or the preservation or the fact that the division of the facial nerve into a hyomandibular and a palatine ramus could have occurred lateral to the braincase. The latter option is more widely observed (LMW, pers. obs.), although detailed information on the peripheral branching of the facial nerve is rare [21]. A possible facial nerve canal in *Falcarius* is only visible in the holotype specimen. Compared to *Erlikosaurus* and *Nothronychus*, it is larger and positioned more rostrally, closer to the trigeminal nerve canal. It concurs with the respective foramina identified by Kirkland et al. [33]. Smith et al. [35] regarded the same foramen as part of the trigeminal foramen. However, the CT data show that the actual trigeminal nerve canal (see above) and this foramen are not connected.

The canal for the vestibulocochlear nerve (CN VIII) originates from the lateral side of the endocast of *Erlikosaurus*, caudal to the facial nerve canal. It leaves the braincase through a small foramen caudal to the foramen for the facial nerve canal (Figure 4A). The vestibulocochlear nerve canal continues a short distance in the caudolateral direction, where it seems to split up into two thin branches. The dorsal branch (vestibular branch) contacts the medial side of the endosseous labyrinth, ventromedial to the ampulla of the caudal semicircular canal. The course of the ventral branch is lost shortly after splitting from the dorsal branch, but it is directed more ventrally and likely represents the cochlear branch. Clark et al. [31] identified a possible additional foramen for the vestibulocochlear nerve, approximately 6 mm dorsal to the foramen described above. However, the respective foramen is connected by a short canal to the base of the crus communis of the endosseous labyrinth, and thus represents the canal for the endolymphatic duct. Its position is consistent with the endolymphatic duct of other coelurosaurs. A further foramen directly rostral to this one, considered by Clark et al. [31] to be the endolymphatic duct canal, cannot be confirmed as such. It is very small and shows no clear trace within the bone and seems to end blind. A foramen or canal for the vestibulocochlear nerve is not visible for *Falcarius* and *Nothronychus* in either the actual specimens or the CT scans.

A large nerve canal on the ventrolateral part of the endocast of *Erlikosaurus* most likely housed the glossopharyngeal (CN IX), the vagus (CN X), and the spinal accessory (CN XI) nerve, as no further nerve canals are visible on the endocast for the separation of these three nerves. The same canal probably also housed the jugular vein. The canal exits the braincase on the medial side through a large and dorsoventrally elongated foramen. The foramen is not divided internally and appears again on the caudal (outside) surface of the braincase between the basioccipital and the exoccipital, lateral to the occipital condyle and the dorsal foramen (details see below) of the hypoglossal nerve. A separate foramen vagi, illustrated by Clark et al. [31], cannot be confirmed in the CT scans. Due to the damage to the braincase on the left side, it is not possible to trace the indicated foramen. On the well preserved right side, this foramen continues in a rostromedial direction, but never connects to the endocast or the preserved cranial nerve canals, but rather ends in a blind pocket ventral to the endosseous labyrinth.

In *Nothronychus* a similar nerve canal originates from the caudolateral side of the endocast and exits the braincase through an enlarged, slit-like foramen lateral to the occipital condyle and two smaller foramina (Figure 5B). Whereas the latter two foramina were described by Kirkland et al. [33] to have contained the vagus and the hypoglossal nerve, this foramen was not described or figured. Neither was it mentioned in the extended description by Smith et al. [35]. Given the close similarity to *Erlikosaurus*, it most likely transmitted the glossopharyngeal, the vagus, and the spinal accessory nerves. The two foramina medial to it therefore housed the two branches of the hypoglossal nerve rather than a separate vagus nerve. Two (or three) hypoglossal foramina are present in several derived theropods [25], [26], [48], [49], [50] and represent a derived condition for Maniraptoriformes [10].

A single large nerve canal is present in the endocasts of *Falcarius*, and is presumed to have housed all three cranial nerves (CN IX–XI). However, as inferred for the troodontid *Zanabazaar junior* [26], the glossopharyngeal nerve might have exited the braincase separately through the fenestra pseudorotunda in *Falcarius,* as well as in *Erlikosaurus* and *Nothronychus*.

The hypoglossal nerve (CN XII) of *Erlikosaurus* is represented by two thin nerve canals, originating on the lower ventrolateral side of the endocast. Both canals are distinctly separated and exit the braincase through two foramina lateral to the occipital condyle on the medial side of the braincase. Although both foramina were illustrated on the inside of the braincase by Clark et al. [31] only one foramen is visible on the outside of the skull. This foramen, through which the more caudal of the two nerve canals exits, is situated in a small depression lateral to the occipital condyle. The respective CT scans show that the rostrally situated canal of the hypoglossal nerve ends approximately 10 mm below the above mentioned foramen on the lower margin of the depression just below the level of the occipital condyle and medial to the joint foramen for cranial nerves IX to XI.

Two distinct nerve canals are also present in the endocast of *Nothronychus*. As discussed above, they probably transmitted the two branches of the hypoglossal nerve instead of single vagus and hypoglossal nerves as suggested by Kirkland et al. [33].

Vascular elements are mostly absent or only weakly indicated on the endocasts of *Erlikosaurus* and *Nothronychus*. The roots of a large and prominent vascular canal are located slightly dorsal and rostral to the floccular lobes on the lateral surface of the cerebellar region (Figure 3A, 5A) of the respective endocasts. They most likely represent the caudal middle cerebral vein. It projects caudally and laterally, but becomes very thin after a few millimeters and seems to drain into the adjacent bone in these taxa. In both endocasts of *Falcarius*, the same venous canal is much more pronounced and extends straight caudally (Figure 6A, 7A). It exits the braincase through an elongate and oval foramen on the lateral surface of the supraoccipital. This foramen is similar in shape and location to that housing the middle cerebral vein in *Majungasaurus* and many other dinosaurs [11], [13], [20], [21]. However, a corresponding foramen is not present in the braincases of *Erlikosaurus* or *Nothronychus*.

Further vascular canals might be concealed within the transverse or dural venous sinuses or not sufficiently appressed against the endocranial wall to leave a groove. Sanders and Smith [51] also noted that the number of foramina increases with the width of the skull and that narrow skulls therefore show fewer foramina. Several of the canals for the cranial nerves might additionally have housed veins and arteries, thus obscuring the real number of vessels.

### Endosseous Labyrinth

**Figure 8:**
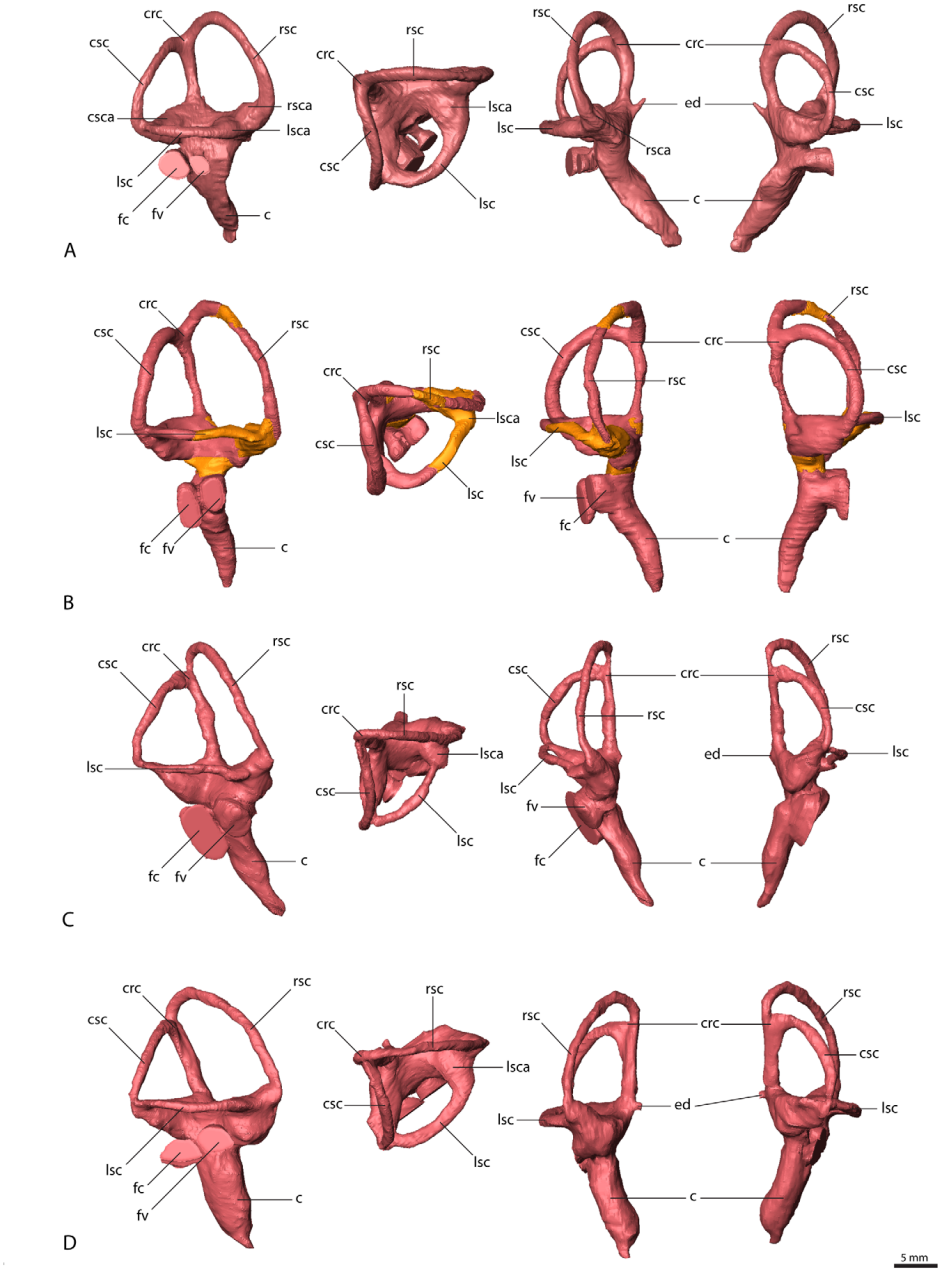
Endosseous labyrinths (right side). (A) *Erlikosaurus andrewsi* (IGM 100/111), (B) *Nothronychus mckinleyi* (AZMNH-2117, portions reconstructed from the left side shown in different color), (C) *Falcarius utahensis* (holotype specimen, UMNH VP 15000), (D) *Falcarius utahensis* (referred specimen, UMNH VP 15001). From left to right in lateral, dorsal, rostral and caudal view. Abbreviations: c, cochlear duct; crc, crus communis; csc, caudal semicircular canal; csca, ampulla of the caudal semicircular canal; fc, fenestra cochleae; ed, endolymphatic duct; fv, fenestra vestibuli; lsc, lateral semicircular canal; lsca, ampulla of the lateral semicircular canal; rsc, rostral semicircular canal; rsca, ampulla of the rostral semicircular canal.

The endosseous labyrinths were extracted for all four therizinosaurian specimens (Figure 8). The labyrinth is most complete and shows the highest level of detail in *Erlikosaurus*. However, due to the damage to the left side of the skull, only the right endosseous labyrinth could be reconstructed. Although the respective regions are slightly less complete and well preserved, the endosseous labyrinths of both sides were reconstructed for *Nothronychus* and the two specimens of *Falcarius* (for details see also Material and methods). Generally, the endosseous labyrinths are similar in their morphology, although there are some distinct inter- and intraspecific differences between the single specimens.

The semicircular canals are thin and delicate in *Erlikosaurus* (Figure 8A). They are oriented nearly perpendicular to each other in the three planes of space. Each of the canals is planar and approximately circular, which gives the dorsal part of the labyrinth a more rectangular outline in lateral view, unlike the triangular shape in many other theropods [18] [20] [21] [52]. The rostral semicircular canal is larger than the other canals and expanded dorsally and rostrally, ascending above the crus communis. Neither the rostral nor the caudal semicircular canal extends beyond the lateral semicircular canal laterally. The ampullae of all three canals are clearly visible and are positioned at the level of the lateral semicircular canal. The crus communis is vertical and straight, without the prominent twisting found in derived maniraptorans and birds 21. At the base of the crus communis, a short endolymphatic duct extends dorsomedially and connects to the endocranial cavity (Figure 4A, B, 8A)

The cochlear duct extends ventromedially from the vestibule in Erlikosaurus. It is elongate and finger-shaped and tapers to a point ventrally. It curves slightly medially, following the outline of the endocast. The fenestra vestibuli and the fenestra cochleae are positioned on the same level below the lateral semicircular canal and face laterally. They have a nearly circular cross-section, with the fenestra vestibuli being slightly smaller. The middle ear cavity is walled off by the prootic and basisphenoid laterally. A small lateral opening between the prootic, the basisphenoid, and the paroccipital process forms the exit of the columellar canal (Figure 4C).

The endosseous labyrinth of *Nothronychus* (Figure 8B) is similar to that of *Erlikosaurus*. The semicircular canals are more oval, which gives the vestibule a more elongate and trapezoidal outline. Otherwise, their orientation is as described for *Erlikosaurus*. The cochlear duct is elongate and thin and more delicate in *Nothronychus*. At its dorsal base are the fenestra vestibuli and fenestra cochleae. Both have a more elliptical cross-section and the fenestra cochleae is considerably enlarged.

Whereas the endosseous labyrinths are similar to each other in *Erlikosaurus* and *Nothronychus*, those in *Falcarius* (Figure 8C, D) are different from these two taxa, and, moreover, the *Falcarius* labyrinths show a considerable degree of variation from each other. Smith et al. [35] presented an interpretive drawing of the left labyrinth of UMNH VP 15001, which only partly reflects the morphology based on our reconstruction. The elongate and thin semicircular canals are expanded dorsally and describe an oval outline, which gives the whole labyrinth a high and triangular shape, which is especially prominent in the holotype specimen (UMNH VP 15000). In both specimens of *Falcarius*, the rostral semicircular canal extends dorsally and caudally beyond the crus communis, whereas the plane of the caudal semicircular canal is angled rostrally. This leads to a slight twisting of the crus communis. The cochlear ducts are thick and prominent and relatively shorter compared to *Erlikosaurus* or *Nothronychus*. The medial curvature of the cochlear ducts is less pronounced in Falcarius, but only UMNH VP 15000 shows a slight rostral expansion. The fenestra vestibuli and the fenestra cochleae are of similar size and have a roughly circular cross-section in UMNH VP 15001, whereas the fenestra cochleae is enlarged and oval in UMNH VP 15000. Witmer and Ridgely [21] attributed some differences in the morphology of endosseous labyrinths of the same species to intraspecific, biological variation and/or slight diagenetic deformation. However, the variation in the two specimens of Falcarius is more prominent than the subtle changes discussed for tyrannosaurs by Witmer and Ridgley [21]. Furthermore, the reconstructions of the left and right labyrinths in both UMNH VP 15000 and UMNH VP 15001 are generally symmetrical, largely ruling out variation caused by deformation. However, fossil elements of Falcarius recovered from the Crystal Geyser Quarry represent a mass death assemblage, with individuals of different sizes and ontogenetic stages [53]. Such anatomical variations in the endosseous labyrinth could thus be the result of different ontogenetic stages or sexual dimorphism, which may be more evident in the endocranial anatomy than in the braincase elements.

### Calculations of Sensory and Cognitive Capabilities

The reconstructed casts of the endocranial features provide the means to calculate linear measurements of the respective structures, thus allowing the rough evaluation of sensory and cognitive capabilities. Olfactory acuity depends both on the absolute size of the olfactory bulbs and their relative size compared to the cerebral hemispheres [30], [54]. Olfactory ratios for *Erlikosaurus* were calculated by measuring the longitudinal and transverse dimensions of the olfactory bulbs and the cerebral hemispheres following Zelenitsky et al. [30]. Olfactory ratio residuals were then produced by correlating the ratio with the respective body mass estimations, producing a range of olfactory ratio residuals. These calculations result in an olfactory ratio of 0.4, and an olfactory ratio residual of 0.01–0.02 for *Erlikosaurus*.

The senses of hearing and balance both reside in the inner ear. The dorsal part of the labyrinth, containing the vestibular apparatus, the semicircular canals, and the otolith organs are associated with the sense of balance, whereas the cochlear part houses the hearing organ. The length of the cochlear ducts has been shown to be closely correlated with hearing frequency sensitivity and auditory capability in extant archosaurs [55]–[57]. Studies by Gleich et al. [58] further demonstrated that body mass can be used to estimate the size of the basilar papilla based on their allometric relationships. Applied to the therizinosaurian specimens in our study, these calculations result in a best frequency of hearing range of 630–1630 Hz and a high frequency hearing limit between 2200 and 4000 Hz (see Table 3).

**Table 3:** Calculations of the best frequency of hearing range and high frequency hearing limits.

	Best frequency of hearing (Hz)	High frequency hearing limit (Hz)
*Erlikosaurus andrewsi*	910–1600	2700–4000
*Nothronychus mckinleyi*	1100–1450	3000–3700
*Falcarius utahensis* (UMNH VP 15000)	630–1630	2200–4000
*Falcarius utahensis* (UMNH VP 15001)	685–1630	2310–4000

Based on [58] (for details see text).

The dimensions and morphology of the vestibular apparatus, and especially the semicircular canals, have been further associated with the senses of balance and equilibrium, as well as locomotor behavior [59], bipedality [60], and the ability to perform rapid head movements. The therizinosaurian specimens in our study follow the general trend in most coelurosaurs in having elongated semicircular canals, with the rostral canal being the longest [21]. A further aspect of the semicircular canals, independent of their sensory implications, is the link between the orientation of the lateral canal and the posture of the head [61], [62]. In what is regarded as the alert posture, the head is aligned with the lateral semicircular canal oriented horizontally. In *Erlikosaurus*, the only therizinosaurian, which preserves an articulated and nearly complete skull, this posture approximates a nearly horizontal position of the head (compare Figure 1, 2A).

The endocast morphology of the investigated specimens provides only a little information regarding the visual capabilities in therizinosaurians. Optic lobes or tecta are not discernible in any of the endocasts and it cannot be determined whether they were located dorsomedially, as in extant reptiles, or ventrolaterally as in extant birds. Further information could be provided by the skull morphology and eyeball dimensions of *Erlikosaurus*. Visual acuity generally depends on eye size [63], [64]. In *Erlikosaurus*, orbital length comprises nearly 25% of the basicranial length of the skull. Although orbit size and shape are only an approximate indicator of eye size [65], [66], it seems plausible that the eyeballs filled the orbital cavities to a large degree. While visual acuity and sensitivity increase with eye size [67], larger eyes do not necessarily have better light-gathering capabilities. This factor is governed to a major part by the corneal diameter [63]. Although only a few sclerotic plates are preserved in *Erlikosaurus* [31], a preliminary reconstruction of the sclerotic ring indicates only a small- to medium-sized aperture (approximately 19–20 mm).

Apart from the morphology and complexity of the brain itself and its single elements, endocast volume has been frequently used to determine cognitive capabilities [10], [28], [29], [68]. Using Avizo’s measuring tools, the volume of the complete endocast (including the olfactory apparatus) can be precisely calculated. These calculations result in a volume of 34.1 cm^3^ and a mass of 35.3 g (assuming a density of 1.036 g/cm^−3^ for brain tissue [61]) for the endocast of *Erlikosaurus*. As a means of calculating and comparing relative brain sizes, a ratio of actual to predicted brain mass, the so-called encephalization quotient (EQ, [68]) is typically employed. This ratio, however, takes into account allometric scaling, and necessitates the knowledge of the respective body mass. Allowing for the range of body-mass estimations (see Material and methods), the reptile encephalization quotient (REQ, a modified version of the EQ to allow for anatomical specifics in reptiles, [44]) of *Erlikosaurus* lies between 2.35 and 3.13. Alternatively, Larsson et al. [28] assessed the ratio between brain and forebrain size to avoid scaling ambiguities and the effect of body mass. Applied to *Erlikosaurus*, the large cerebral hemispheres comprise more than 40% of the endocast.

## Discussion

### Sensorineural Implications

The sensorineural calculations for the endocasts under study here indicate that the sensory capabilities were relatively high in therizinosaurians. In particular, olfaction was well developed in at least *Erlikosaurus*, where the olfactory ratios and residuals are higher than predicted given its body mass. Zelenitsky et al. [30], [54] suggested that the lower than expected olfactory values for ornithomimosaurs and oviraptorids in their study reflect adaptations to an omnivorous diet, whereas predators such as tyrannosaurs have higher than expected values. Thus, given that therizinosaurians are widely regarded as herbivorous [2], [5], the presence of unreduced olfactory capabilities in *Erlikosaurus* (and probably therizinosaurians in general based on incomplete elements in *Falcarius*; see above) is surprising and could mean two things: (1) moderate to high olfactory abilities played an important role in foraging or (2) relatively large olfactory ratios are an ancestral feature retained in this group. The Aptian and Albian are characterized by the radiation of flowering plants [69], [70]. Angiosperm leaves, fruits, and flowers became more abundant and are presumed to have been highly odoriferous [71]. Olfactory acuity could thus have been a crucial factor to discriminate between digestible and indigestible plants – an essential prerequisite to keep up with metabolic and energetic (endothermic) demands [72]. Thus, even if exceptional olfactory abilities did not evolve directly as a response to a dietary transition, the retention of these (plesiomorphic) sensory capabilities would still prove to be ecologically beneficial.

A similar pattern can be found in the auditory capabilities. Our results indicate that therizinosaurians were able to discriminate a comparably wide range of frequencies in a low to middle frequency domain. The auditory capabilities were similar or even higher in these taxa than in other herbivorous dinosaur groups, such as ornithischians [13], [14] or sauropods [11], but generally comparable to tyrannosaurs [21] suggesting an at least partly phylogenetic rather than purely ecological signal. Walsh et al. [57] found a positive correlation between cochlear duct length and sociality and environmental complexity in their study, whereas habitat did not seem to have a significant influence on hearing. However, the palaeoenvironment of the Cretaceous contained a plethora of acoustic signals, made by a variety of insects, small tetrapods, birds, and other dinosaurs [73], [74]. The auditory and frequency sensitivity in therizinosaurians could therefore have been an apomorphic or ancestral adaptation to dietary specializations, intraspecific sociality, or predator evasion.

Regarding the visual abilities in therizinosaurians, the lack of prominent and ventrolaterally displaced optic tecta would suggest that their visual capabilities were only low to moderately developed. However, due to equivocal data in tyrannosaurs, Witmer and Ridgely [21] suggested that ventrolaterally displaced optic tecta might have been present in tyrannosaurs, but concealed by a large dural envelope. The same could be true for the optic lobes of *Erlikosaurus*, showing a transitional stage in the evolution of a more birdlike brain.

Schmitz and Motani [75] inferred visual sensitivity and daily activity patterns for several non-avian theropods based on orbit and sclerotic ring diameters. Their samples included no therizinosaurians, but, applied to our data, their findings would indicate that *Erlikosaurus* had probably a lower visual sensitivity than other theropods. *Ornithomimus edmonticus* and *Garudimimus brevipes*, as well as most herbivores in their study [75], were found to be mesopic, whereas most predators were scored as scotopic. Given the probably low visual sensitivity and light-gathering capabilities, but higher visual acuity, it seems likely that *Erlikosaurus* was photopic. Still, the enlarged floccular lobes and elongated semicircular canals indicate that vision was at least moderately developed in terms of coordination between eye and head movement. Witmer and Ridgely [21] explained the expression of these features with the adaptations for bipedality in tyrannosaurs and other coelurosaurs, but also suggested a functional role of the semicircular canals in gaze stabilization and eye movement. Based on recent studies [61], [76], they have shown that neural links between the semicircular canals, the floccular lobes, and the ocular musculature are responsible in detecting and coordinating movement of the eyes, neck, and head. Given the relatively large size of the floccular lobes in *Erlikosaurus* and *Nothronychus*, it can be assumed that these taxa were capable of keeping their gaze focused on a target during rapid head and neck movements–a requirement typically associated with predation and/or flight. Alternatively, refined gaze stabilization mechanisms might have been advantageous for other behaviors (e.g., territoriality, courtship) or may simply be plesiomorphic reflections of their coelurosaurian heritage. In this context, it is worth noting that extant flightless herbivorous birds such as ostrich (*Struthio camelus*) likewise retain elongate semicircular canals.

Inferred from the position of the lateral semicircular canal, *Erlikosaurus* would have had a nearly horizontal head posture, which could be linked to allow an optimal overlap of the visual fields and binocular vision. The low cephalic and pontine flexure of the brain observed in *Erlikosaurus* might thus be an adaptation to the osteological requirements in keeping a horizontal deportment of the head and upper neck [77]. Very little comparative information on brain angulation is available, but high values of pontine and cephalic flexure are observed in basal theropods, as well as in most maniraptorans and birds [10], [20], [21], [29]. *Struthiomimus altus* in contrast, has a relatively straight endocast similar to that of *Erlikosaurus*. Low pontine flexure is also present in *Nothronychus* and the referred specimen of *Falcarius* (UMNH VP 15001), whereas the holotype specimen of *Falcarius* (UMNH VP 15000) shows a pronounced pontine flexure. Although the forebrain and partly the midbrain regions in the latter taxa are unknown, the amounts of pontine and cephalic flexure are generally directly correlated in dinosaurs [78]. Similar to the variations observed in the inner ear morphology, the obvious discrepancy in the pontine and cephalic flexure between the two specimens of *Falcarius* could be explained by different ontogenetic stages, as the holotype specimen most likely represent a juvenile individual [43], which tend to have a more highly flexed endocast than adults [75], due to the fact that brain and skull growth proceed at different rates [79].

### Cognitive Inferences

**Figure 9:**
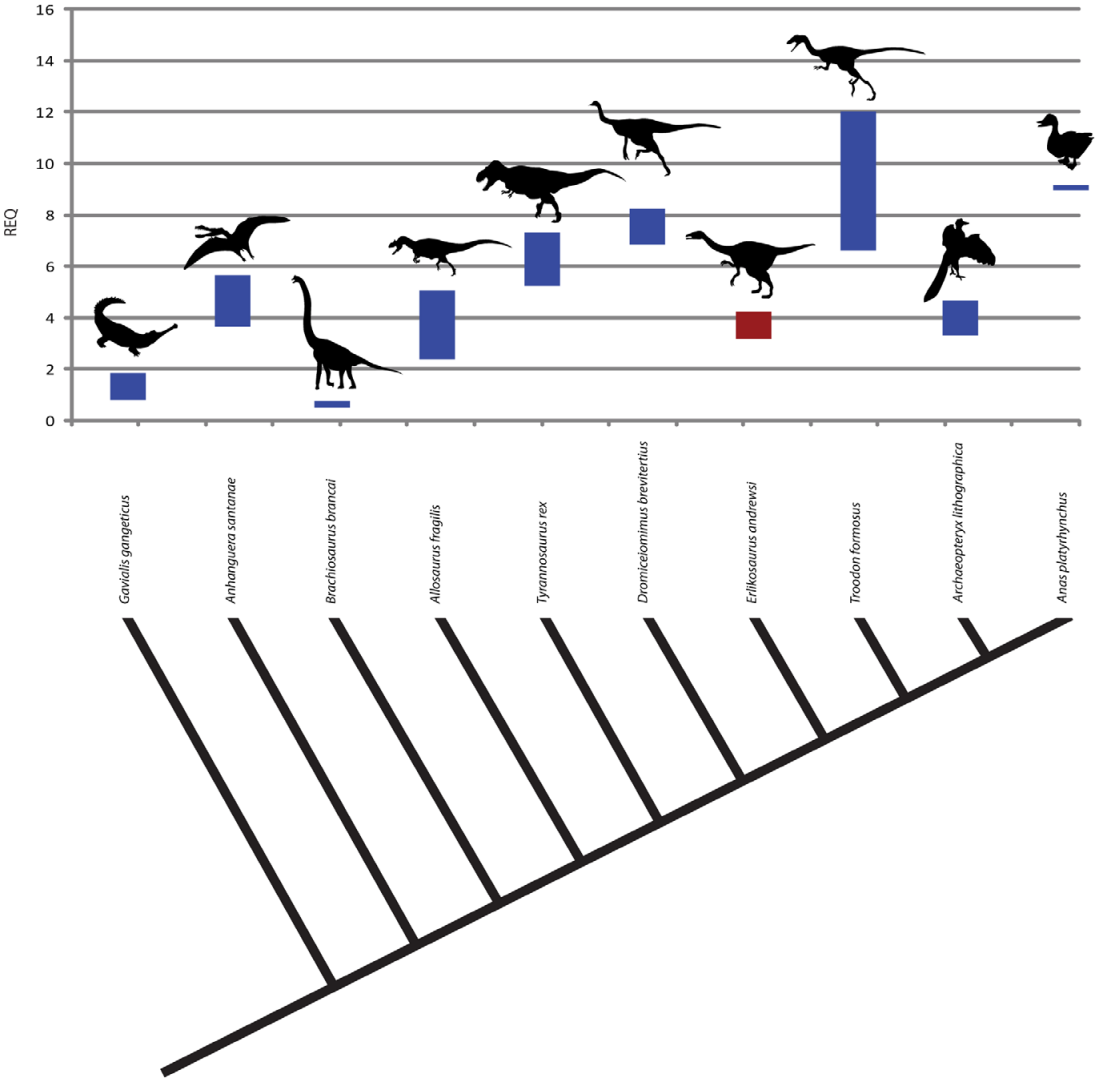
Reptile encephalization quotient of *Erlikosaurus andrewsi* in comparison to several extant and extinct archosaurs. Supplemented by data from [10], [29].

Wharton [29] and Franzosa [10] independently collected a wide range of REQs for extant and extinct archosaurs. Compared to their results, the REQ of *Erlikosaurus* falls within the range of basal theropods and coelurosaurs (*Ceratosaurus magnicornis,* 3.31–5.17, *Allosaurus fragilis,* 2.44–5.24; *Carcharodontosaurus saharicus* 2.3–3.23) (Figure 9). Although only a few values for the REQ in maniraptoriform taxa are available, they are significantly higher than in *Erlikosaurus* (*Dromiceiomimus brevitertius*, 7.15–8.61; *Troodon formosus*, 6.89–12.53), with the exception of *Archaeopteryx lithographica* (3.41–4.85), which shows only a slightly higher ratio.

Considering the large uncertainties in body mass, allometric factors, and the extent to which the actual brain filled the endocranial cavity, Larsson et al. [28] assessed the ratio between brain and forebrain, represented by the cerebral hemispheres. Based on the findings for *Tyrannosaurus rex* (32.6%) and *Archaeopteryx lithographica* (44.6%), they assumed an intermediate stage of forebrain enlargement was present in derived maniraptorans, such as *Troodon* and *Caudipteryx*. The value for *Erlikosaurus* also falls within that range, indicating that forebrain enlargement must have occurred very close to the base of Maniraptora.

### Conclusions

This study presents the first reconstruction and visualization of the endocranial anatomy of therizinosaurians and sheds light on the sensory adaptations within this group. Phylogenetically positioned between basal coelurosaurs and derived maniraptoran dinosaurs, the brain anatomy of therizinosaurians represents a mixture between plesiomorphic and derived characteristics. Although situated near the base of Maniraptora, therizinosaurians had developed the neural pathways for a well-developed sensory repertoire. The anatomy of the olfactory apparatus and the endosseous labyrinth suggests that olfaction, hearing, and equilibrium were well developed in therizinosaurians and might have affected or benefited from an enlarged telencephalon. The high acuity of the individual senses further indicates that they may have played an important role in foraging, predator evasion, and/or social complexity. However, it is important to note that, although a variety of osteological adaptations provide good evidence for derived dietary preferences in Therizinosauria, the sensory abilities could constitute a retention of plesiomorphic traits, rather than a direct result of an ecological transformation. Considering the wider distribution of these sensory and neural characteristics among basal coelurosaurs, therizinosaurians could thus simply have utilized an already existing sensory repertoire inherited from their carnivorous ancestors for different dietary strategies. Further investigations of sensory capabilities in Maniraptora are needed to differentiate between phylogenetic and ecological signals in the evolution of these traits.

## Supporting Information

Figure S1 **Interactive figure of the cranial endocast of** ***Erlikosaurus andrewsi*** **(IGM 100/111).**
(PDF)

Figure S2 **Interactive figure of the cranial endocast and braincase of** ***Nothronychus mckinleyi*** **(AZMNH-2117).**
(PDF)

Figure S3 **Interactive figure of the cranial endocast and braincase of** ***Falcarius utahensis*** **(holotype, UMNH VP 15000).**
(PDF)

Figure S4 **Interactive figure of the cranial endocast and braincase of** ***Falcarius utahensis*** **(referred specimen, UMNH VP 15001).**
(PDF)

Figure S5 **Interactive figure of the endosseous labyrinths (right side) of** ***Erlikosaurus andrewsi*** **(IGM 100/111),** ***Nothronychus mckinleyi*** **(AZMNH-2117), and** ***Falcarius utahensis*** **(UMNH VP 15000, UMNH VP 15001).**
(PDF)
