# Supplementary material for: The Endocranial Anatomy of Therizinosauria and Its Implications for Sensory and Cognitive Function
Source: PLoS One. 2012 Dec 19;7(12):e52289. doi: 10.1371/journal.pone.0052289 (PMC3526574; doi:10.1371/journal.pone.0052289)
Supplement: Figure S1 — Interactive figure of the cranial endocast of Erlikosaurus andrewsi (IGM 100/111). (PDF) [file pone.0052289.s001.pdf]

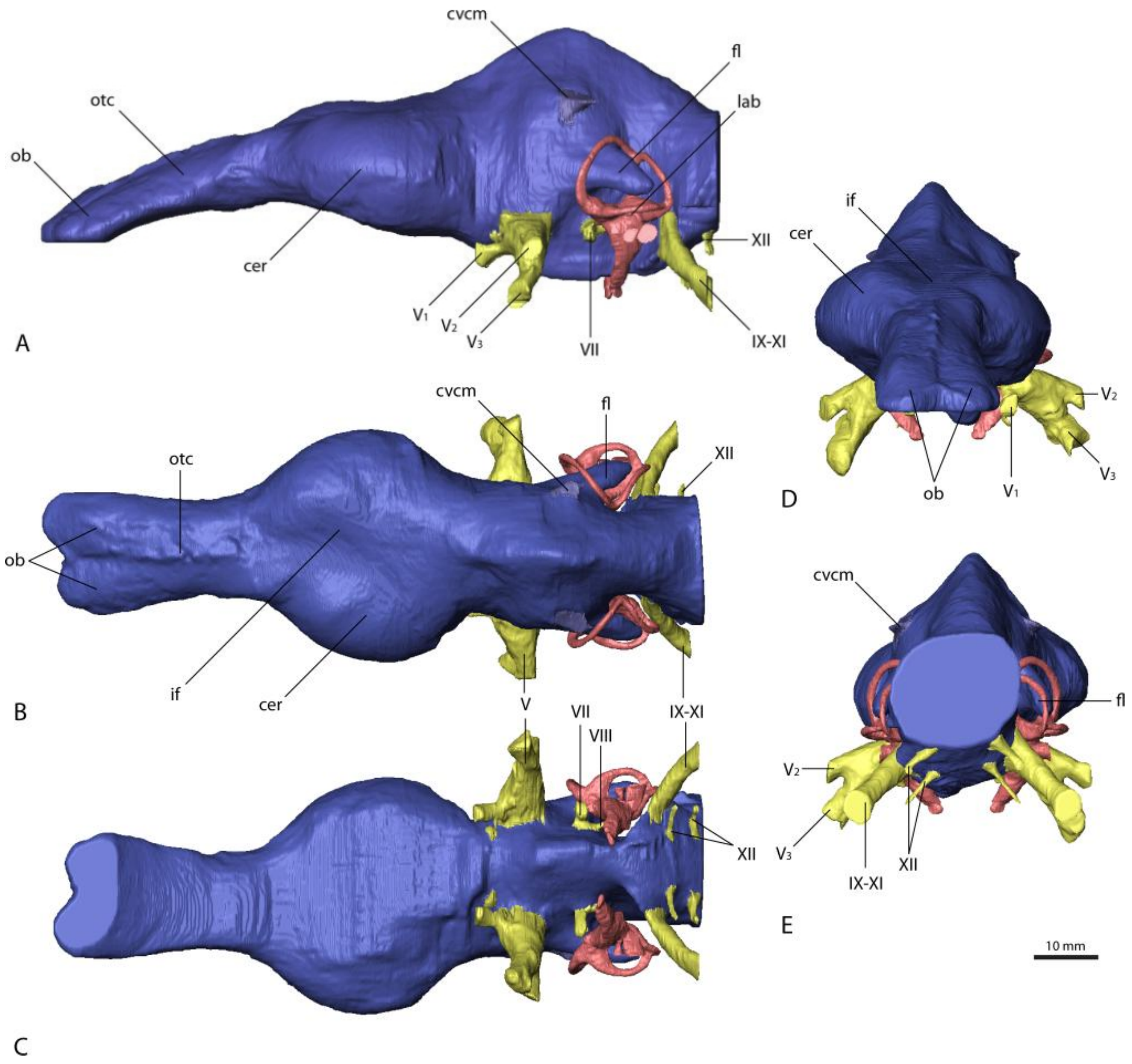

Figure S1. Cranial endocast of *Erlikosaurus andrewsi* (IGM 100/111). In (A) left lateral, (B) dorsal, (C) ventral, (D) rostral, and (E) caudal view. Abbreviations: cer, cerebral hemisphere; cvcm, caudal middle cerebral vein; fl, floccular lobe; if, interhemispherical fissure; lab, endosseous labyrinth; ob, olfactory bulbs; otc, olfactory tracts; V_1, ophthalmic branch of the trigeminal nerve canal; V_2, maxillary branch of the trigeminal nerve canal; V_3, mandibular branch of the trigeminal nerve canal; VII, facial nerve canal; VIII, vestibulocochlear nerve canal; IX-XI, shared canal for the glossopharyngeal, vagus and spinal accessory nerve; XII, hypoglossal nerve canal. 3D content can be activated by clicking on figure S1 A.
