# Supplementary material for: The Endocranial Anatomy of Therizinosauria and Its Implications for Sensory and Cognitive Function
Source: PLoS One. 2012 Dec 19;7(12):e52289. doi: 10.1371/journal.pone.0052289 (PMC3526574; doi:10.1371/journal.pone.0052289)
Supplement: Figure S2 — Interactive figure of the cranial endocast and braincase of Nothronychus mckinleyi (AZMNH-2117). (PDF) [file pone.0052289.s002.pdf]

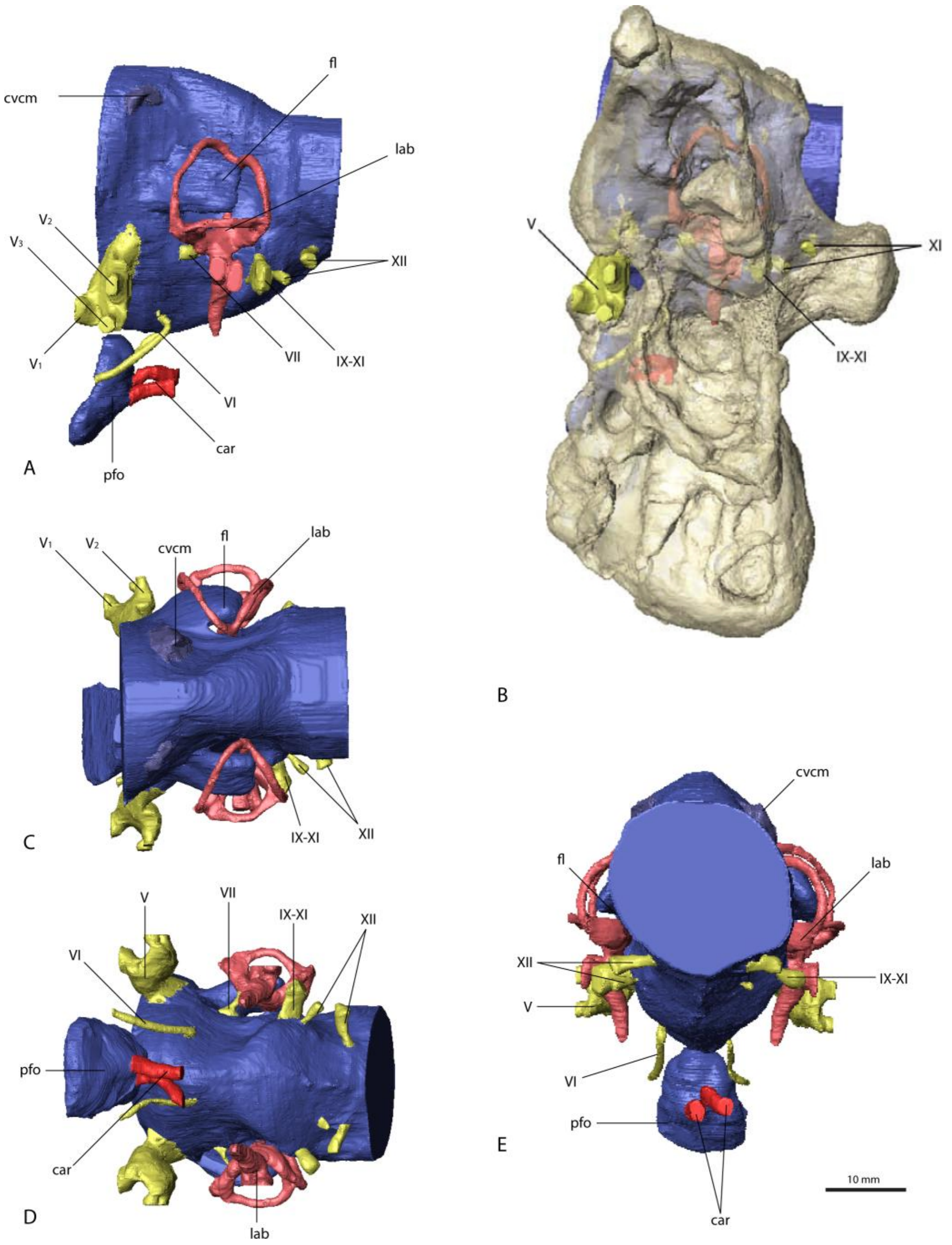

Figure S2. Cranial endocast and braincase of *Nothronychus mckinleyi* (AZMNH-2117). In (A) and (B) in left lateral, (C) caudal, (D) dorsal, and (E) ventral view. Bone in (B) rendered transparent. Abbreviations: car, cerebral carotid artery canal; cvcm, caudal middle cerebral vein; fl, floccular lobe; lab, endosseous labyrinth; pfo, pituitary (hypophyseal) fossa; V_1, ophthalmic branch of the trigeminal

nerve canal; V_2, maxillary branch of the trigeminal nerve canal; V_3, mandibular branch of the trigeminal nerve canal; VI, abducens nerve canal; VII, facial nerve canal; IX-XI, shared canal for the glossopharyngeal, vagus and spinal accessory nerve; XII, hypoglossal nerve canal. 3D content can be activated by clicking on figure S2 B.
