# Supplementary material for: The Endocranial Anatomy of Therizinosauria and Its Implications for Sensory and Cognitive Function
Source: PLoS One. 2012 Dec 19;7(12):e52289. doi: 10.1371/journal.pone.0052289 (PMC3526574; doi:10.1371/journal.pone.0052289)
Supplement: Figure S3 — Interactive figure of the cranial endocast and braincase of Falcarius utahensis (holotype, UMNH VP 15000). (PDF) [file pone.0052289.s003.pdf]

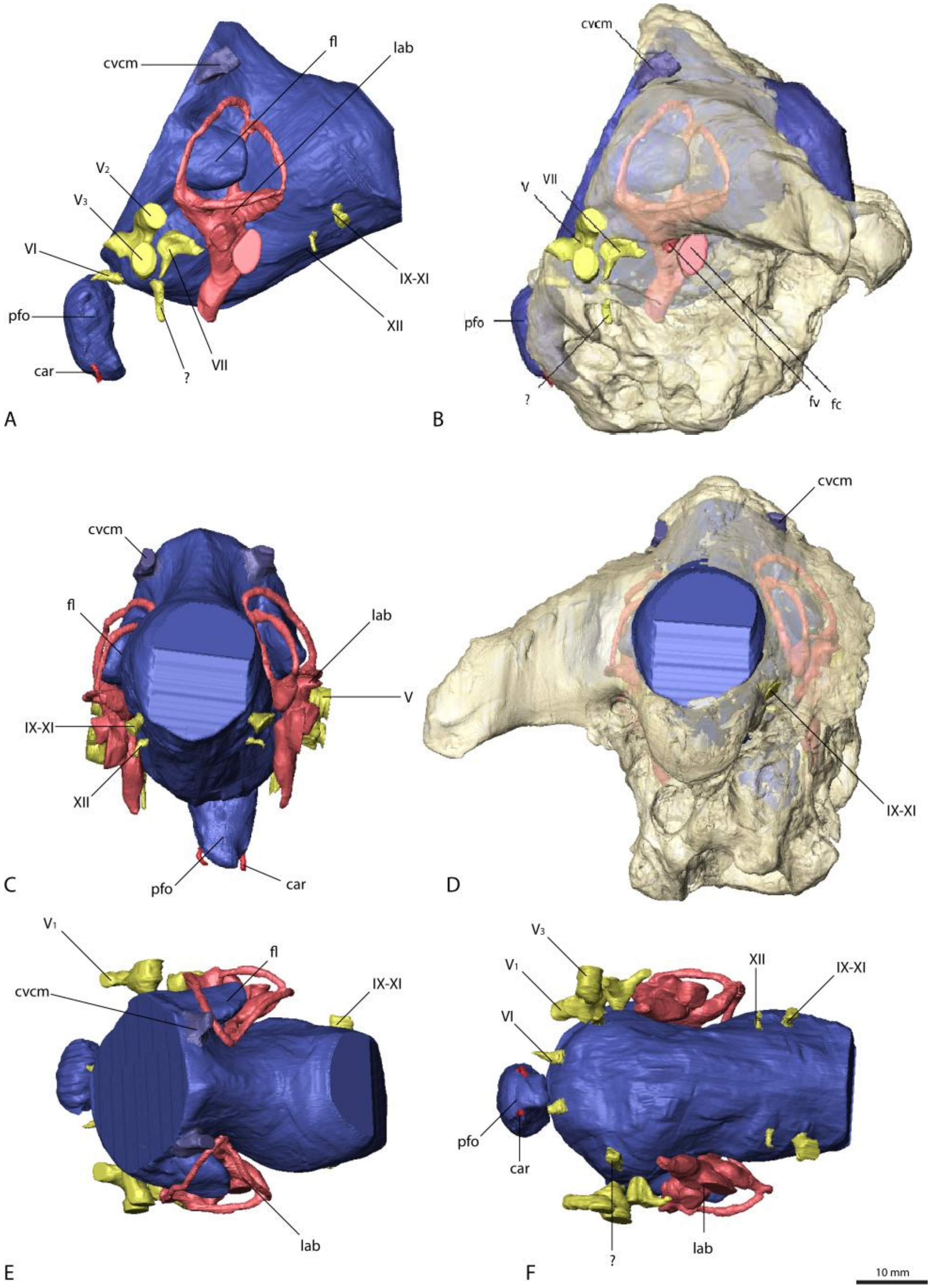


**Figure S3. Cranial endocast and braincase of *Falcarius utahensis* (holotype, UMNH VP 15000).**

In (A) and (B) in left lateral, (C) and (D) in caudal, (E) dorsal, and (F) ventral view. Bone in (B) and

(D) rendered transparent. Abbreviations: car, cerebral carotid artery canal; cvcm, caudal middle cerebral vein; fc, fenestra cochleae; fl, floccular lobe; fv, fenestra vestibuli; lab, endosseous labyrinth; pfo, pituitary (hypophyseal) fossa; $V_1$, ophthalmic branch of the trigeminal nerve canal; $V_2$, maxillary branch of the trigeminal nerve canal; $V_3$, mandibular branch of the trigeminal nerve canal; VI, abducens nerve canal; VII, facial nerve canal; IX-XI, shared canal for the glossopharyngeal, vagus and spinal accessory nerve; XII, hypoglossal nerve canal. 3D content can be activated by clicking on figure S3 B.
