# Supplementary material for: The Endocranial Anatomy of Therizinosauria and Its Implications for Sensory and Cognitive Function
Source: PLoS One. 2012 Dec 19;7(12):e52289. doi: 10.1371/journal.pone.0052289 (PMC3526574; doi:10.1371/journal.pone.0052289)
Supplement: Figure S4 — Interactive figure of the cranial endocast and braincase of Falcarius utahensis (referred specimen, UMNH VP 15001). (PDF) [file pone.0052289.s004.pdf]

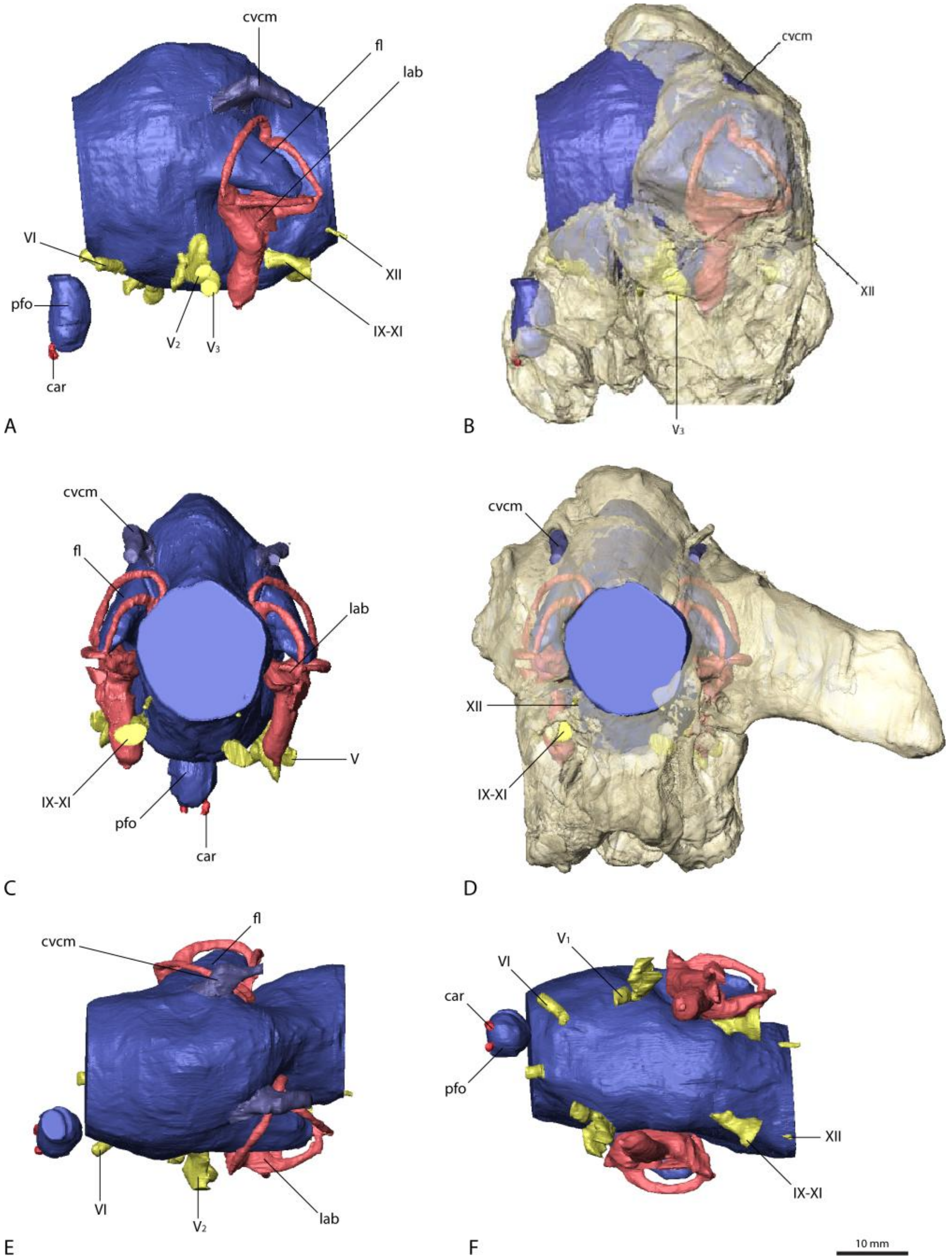

Figure S4. Cranial endocast and braincase of *Falcarius utahensis* (referred specimen, UMNH VP 15001). In (A) and (B) in left lateral, (C) and (D) in caudal, (E) dorsal, and (F) ventral view. Bone in (B) and (D) rendered transparent. Abbreviations: car, cerebral carotid artery canal; cvcm, caudal

middle cerebral vein; fl, floccular lobe; lab, endosseous labyrinth; pfo, pituitary (hypophyseal) fossa; V_1, ophthalmic branch of the trigeminal nerve canal; V_2, maxillary branch of the trigeminal nerve canal; V_3, mandibular branch of the trigeminal nerve canal; VI, abducens nerve canal; IX-XI, shared canal for the glossopharyngeal, vagus and spinal accessory nerve; XII, hypoglossal nerve canal. 3D content can be activated by clicking on figure S4 B.
