# Supplementary material for: The Endocranial Anatomy of Therizinosauria and Its Implications for Sensory and Cognitive Function
Source: PLoS One. 2012 Dec 19;7(12):e52289. doi: 10.1371/journal.pone.0052289 (PMC3526574; doi:10.1371/journal.pone.0052289)
Supplement: Figure S5 — Interactive figure of the endosseous labyrinths (right side) of Erlikosaurus andrewsi (IGM 100/111), Nothronychus mckinleyi (AZMNH-2117), and Falcarius utahensis (UMNH VP 15000, UMNH VP 15001). (PDF) [file pone.0052289.s005.pdf]

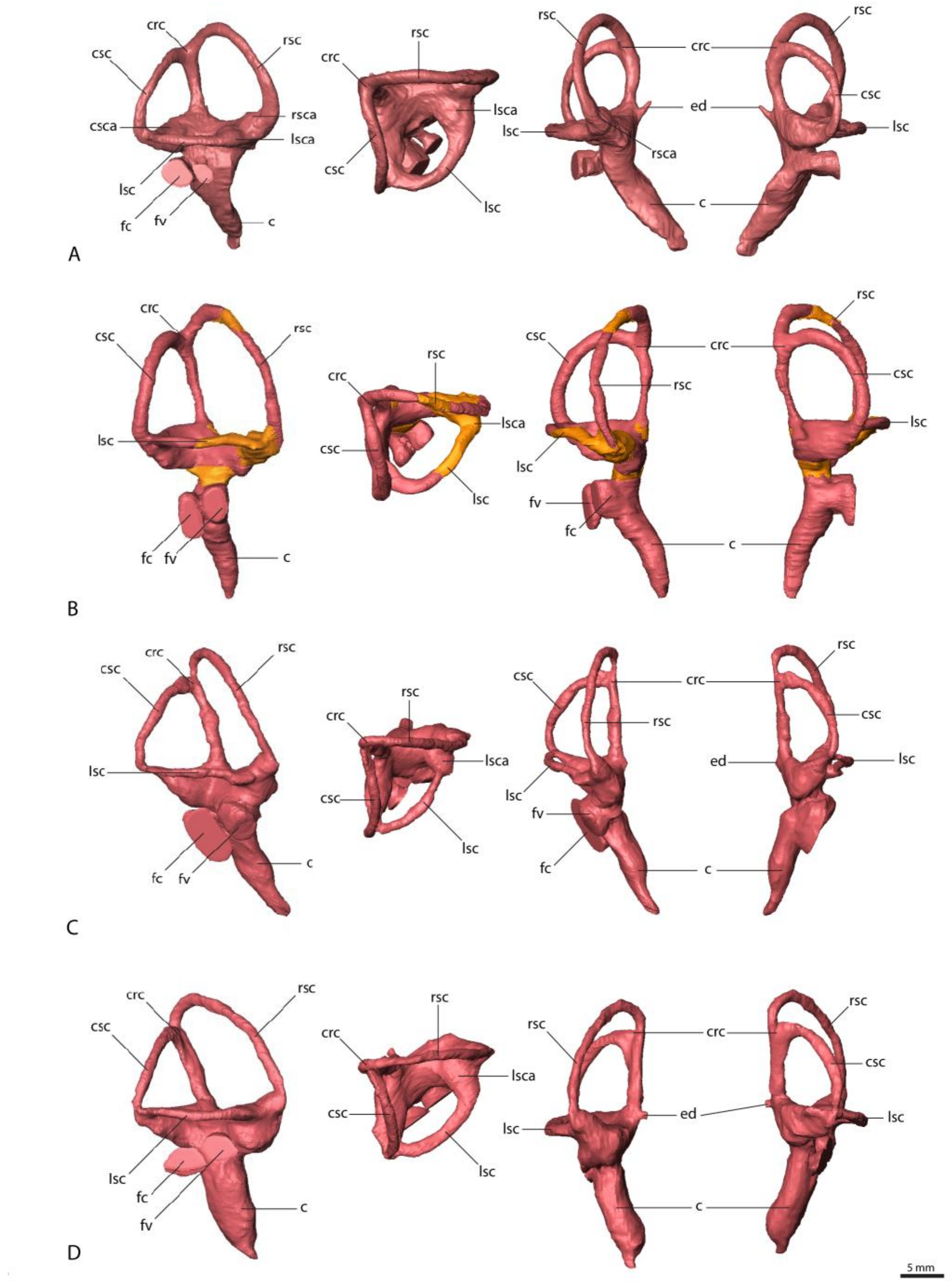

Figure S5. Endosseous labyrinths (right side). (A) *Erlikosaurus andrewsi* (IGM 100/111), (B) *Nothronychus mckinleyi* (AZMNH-2117, portions reconstructed from the left side shown in different color), (C) *Falcarius utahensis* (holotype specimen, UMNH VP 15000), (D) *Falcarius utahensis* (referred specimen, UMNH VP 15001). From left to right in lateral, dorsal, rostral and caudal view.

Abbreviations: c, cochlear duct; crc, crus communis; csc, caudal semicircular canal; csca, ampulla of the caudal semicircular canal; fc, fenestra cochleae; ed, endolymphatic duct; fv, fenestra vestibuli; lsc, lateral semicircular canal; lsca, ampulla of the lateral semicircular canal; rsc, rostral semicircular canal; rsca, ampulla of the rostral semicircular canal. 3D content can be activated by clicking on figure S5 A-D.
